# ProMotE: an efficient algorithm for counting independent motifs in uncertain network topologies

**DOI:** 10.1186/s12859-018-2236-9

**Published:** 2018-06-26

**Authors:** Yuanfang Ren, Aisharjya Sarkar, Tamer Kahveci

**Affiliations:** Department of Computer & Information Science & Engineering, University of Florida, Gainesville, 32611 FL USA

**Keywords:** Independent motif counting, Probabilistic networks, Polynomial

## Abstract

**Background:**

Identifying motifs in biological networks is essential in uncovering key functions served by these networks. Finding non-overlapping motif instances is however a computationally challenging task. The fact that biological interactions are uncertain events further complicates the problem, as it makes the existence of an embedding of a given motif an uncertain event as well.

**Results:**

In this paper, we develop a novel method, *ProMotE* (**Pro**babilistic **Mot**if **E**mbedding), to count non-overlapping embeddings of a given motif in probabilistic networks. We utilize a polynomial model to capture the uncertainty. We develop three strategies to scale our algorithm to large networks.

**Conclusions:**

Our experiments demonstrate that our method scales to large networks in practical time with high accuracy where existing methods fail. Moreover, our experiments on cancer and degenerative disease networks show that our method helps in uncovering key functional characteristics of biological networks.

## Background

Biological networks describe a system of interacting molecules. Through these interactions, these molecules carry out key functions such as regulation of transcription and transmission of signals [1]. Biological networks are often modeled as graphs, with nodes and edges representing interacting molecules (e.g., protein or gene) and the interactions between them respectively [2–4]. Studying biological networks has great potential to provide significant new insights into systems biology [5, 6].

Network motifs are patterns of local interconnections occurring significantly more in a given network than in a random network of the same size [7]. Identifying motifs is crucial to uncover important properties of biological networks. They have already been successfully used in many applications, such as understanding important genes that affect the spread of infectious diseases [8], revealing relationship across species [6, 9], and discovering processes which regulate transcription [10].

Network motif discovery is a computationally hard problem as it requires solving the well-known subgraph isomorphism problem, which is NP-complete [11]. The fact that biological interactions are often inherently stochastic events further complicates the problem [12]. An interaction may or may not happen with some probability. This uncertainty follows from the fact that biological processes governing these interactions, such as DNA replication process, inherently exhibit uncertainties. For example, DNA replication can initiate at different chromosome locations with various probabilities [13]. Besides the replication time variance, other epigenetic factors can also alter the expression levels of genes, which in turn affect the ability of proteins to interact [14].

Existing studies model the uncertainty of biological interactions using a probability value showing the confidence in its presence [12]. More specially, each edge in the network is associated with a probability value. Several databases, such as MINT [15] and STRING [16], already provide interaction confidence values. If a biological network has at least one uncertain interaction, we call it a probabilistic network. Otherwise, it is a *deterministic network*. In the rest of the paper, we represent a probabilistic network using a graph denoted with *G*=(*V,E,P*), where *V* denotes the set of interacting molecules, *E* denotes the interactions among them, and *P*:*E*→(0,1] is the function that assigns a probability value to each edge.

Several approaches have been developed to solve the network motif discovery problem (e.g., [17–19]). However, most of them focus on deterministic network topologies. The main reason behind this limitation is that a probabilistic network summarizes all deterministic networks generated by all possible subsets of interactions. Thus, a probabilistic network *G*=(*V,E,P*) yields 2^|*E*|^ deterministic instances. The exponential growth of the number of deterministic instances makes it impossible to directly apply existing solutions to probabilistic networks. Relatively little research has been done on finding motifs in probabilistic networks. Tran et al. [20] proposed a method to derive a set of formulas for count estimation. This study however has not provided a general mathematical formulation for arbitrary motif topologies. It rather requires a unique mathematical formulation for each motif. Besides, it assumes that all interactions of the probabilistic network have the same probability. Thus, it fails to solve the generalized version of the problem where each interaction takes place with a possibly different probability. Todor et al. [21] developed a method to solve the generalized version of the problem. It computes the exact mean and variance of the number of motif instances. Both of above two methods count the maximum number of motif instances using $\mathcal {F}_{1}$ measure, that is including all possible embeddings regardless of whether they overlap with each other or not.

**Fig. 1:**
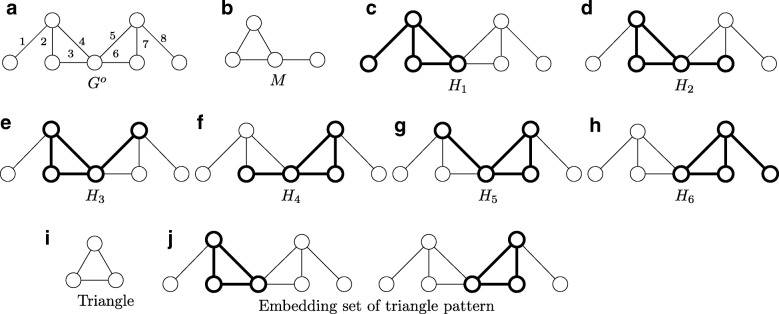
An example to explain three frequency measures. **a** A hypothetical deterministic network *G*^*o*^ with seven nodes and eight edges. **b** A motif pattern *M* with four nodes and three edges. **c** - **h** Six possible embeddings of motif pattern *M* in network *G*^*o*^ denoted with the embedding set $\mathcal {H} =\{H_{1}, H_{2}, H_{3}, H_{4}, H_{5}, H_{6}\}$. **i** A triangle pattern. **j** An embedding set of triangle pattern

There are two more restrictive frequency measures, $\mathcal {F}_{2}$ and $\mathcal {F}_{3}$, which avoid reuse of graph elements [19]. $\mathcal {F}_{2}$ measure considers that two embeddings of a motif *overlap* if they share an edge. $\mathcal {F}_{3}$ measure is more restrictive as it defines overlap as sharing of a node. These two measures count the maximum number of *non-overlapping* embeddings of a given motif. We explain the difference among three frequency measures on a hypothetical deterministic network *G*^*o*^ (see Fig. 1a). Consider the motif pattern *M* in Fig. 1b. *G*^*o*^ yields six possible embeddings of *M* denoted with the embedding set $\mathcal {H} = \{H_{1},H_{2},H_{3}, H_{4}, H_{5}, H_{6}\}$ (see Fig. 1c-h). Since $\mathcal {F}_{1}$ measure counts all possible embeddings, the $\mathcal {F}_{1}$ count is six. As embeddings *H*_1_ and *H*_6_ do not have common edges, the $\mathcal {F}_{2}$ count is two. All pairs of embeddings in this set share nodes. As a result, the $\mathcal {F}_{3}$ count is one.

$\mathcal {F}_{2}$ and $\mathcal {F}_{3}$ measures satisfy a fundamental characteristic, the *downward closure property*, which $\mathcal {F}_{1}$ measure fails to have. This property is essential for constructing large motifs [22]. It ensures that the frequency of network motifs is monotonically decreasing with increasing size of motif patterns. For example, in the deterministic network *G*^*o*^ (see Fig. 1a), given the triangle pattern (see Fig. 1i), there are two triangle embeddings in total (Fig. 1j). Consider a larger motif pattern, such as the pattern in Fig. 1b. The $\mathcal {F}_{1}$ count however becomes six, which conflicts with the downward closure property. Besides, non-overlapping motifs are needed in navigation methods such as folding and unfolding of the network [23]. Taking the importance of non-overlapping motifs into account, Sarkar et al. [24] developed a method to count the non-overlapping motifs in probabilistic networks using the $\mathcal {F}_{2}$ measure. Their study builds a polynomial to model the distribution of the number of motif instances overlapping with a specific embedding of that motif. However, the exponential growth of the size of polynomial terms makes it not scalable to large networks.

**Contributions.** In this paper, we develop a scalable method, named *ProMotE* (**Pro**babilistic **Mot**if **E**mbedding), to tackle the problem of counting independent motifs in a given probabilistic network. We formally define the problem in “Preliminaries and problem definition” section. We explain our method for the $\mathcal {F}_{2}$ measure, yet the same algorithm can trivially be applied to the $\mathcal {F}_{3}$ measure. This study has three major contributions over the existing literature: (1) The key bottleneck in counting motifs in probabilistic networks is computing the distribution of the number of overlapping embeddings of a given motif instance. We build a new method which allows us to avoid computing this distribution whenever possible. (2) Computing the distribution in (1) above necessitates constructing a polynomial. We devise two strategies, which compute bounds to the overlapping motif count distribution prior to constructing the entire polynomial. These bounds enable us to terminate the costly computation of the distribution whenever possible. (3) We develop a new strategy which allows multiplication of arbitrarily large polynomials using a limited amount of memory. Our experimental results demonstrate that our algorithm is orders of magnitude faster than existing methods. Our results on cancer and disease networks suggest that our method can help in uncovering key functional characteristics of the genes participating in those networks.

We organize the rest of the paper as follows. We present our algorithm in “Methods” section. We discuss our experimental results in “Results and discussion” section and conclude in “Conclusions” section.

## Methods

In this section, we present our method, ProMotE. First, we formally define the independent motif counting problem in probabilistic networks (“Preliminaries and problem definition” section). We next summarize the method by Sarkar et al. [24] (“Overview of the existing solution” section). We then present the method developed in this paper. Our method introduces three strategies (Sections “Avoiding loss computation”, “Efficient polynomial collapsation” and “Overcoming memory bottleneck”), which help us scale to large network size, for which existing methods fail.

### Preliminaries and problem definition

**Fig. 2:**
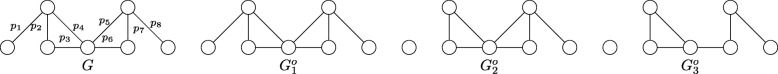
A probabilistic network *G* and three of its possible deterministic network topologies denoted with $G_{1}^{o}$, $G_{2}^{o}$ and $G_{3}^{o}$

In this section we present basic notation needed to define the problem considered in this paper. We denote the given probabilistic network and motif pattern with *G*=(*V,E,P*) and *M* respectively. For each edge *e*_*i*_ in *G*, we denote the probability that *e*_*i*_ is present and absent with *p*_*i*_ and *q*_*i*_ respectively (i.e., *p*_*i*_+*q*_*i*_=1). We denote the set of all possible deterministic network topologies one can observe from *G* with $ \mathcal {D}(G) = \{G^{o} = (V, E^{o}) | ~E^{o} \subseteq E\}$. We denote a specific deterministic network which inherits all nodes and edges from *G* but assume that all of its edges exist with *G*^′^=(*V*,*E*). Figure 2 depicts a probabilistic network and its three possible deterministic networks (i.e., in total there are 2^8^=256 deterministic networks). We denote the probability of observing a specific deterministic network $G^{o} \in \mathcal {D}(G)$ with

$$\mathcal{P}(G^{o}|G) = \prod_{e_{i} \in E^{o}}p_{i} \prod_{e_{j} \in E-E^{o}}q_{j}.$$

**Fig. 3:**
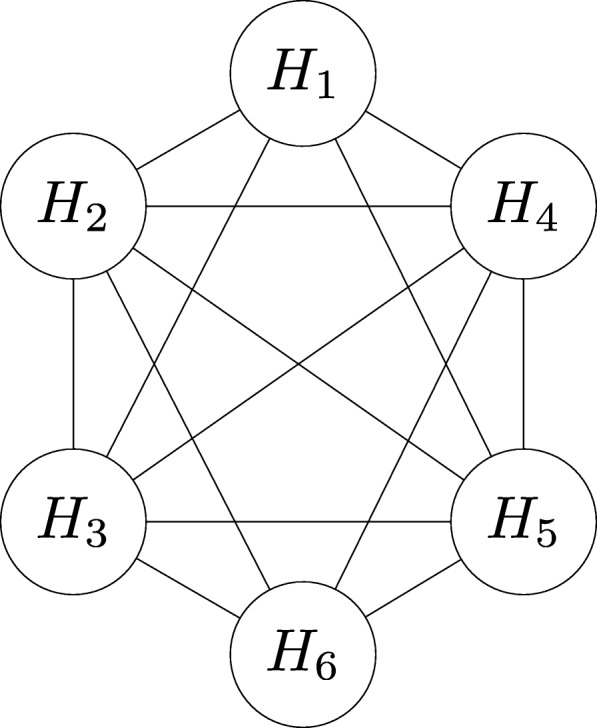
The overlap graph $\bar {G^{o}}$ of the deterministic network *G*^*o*^ (Fig. 1a) for its six embeddings (Fig. 1c-h)

Given a deterministic network *G*^*o*^=(*V*,*E*^*o*^) and a motif pattern *M*, we represent the set of all its embeddings with $\mathcal {H}(M|G^{o})$. We construct the *overlap graph* for $\mathcal {H}(M|G^{o})$, denoted with $\bar {G^{o}}$, by representing each embedding $H_{k} \in \mathcal {H}(M|G^{o})$ as a node and inserting an edge into two nodes if their corresponding embeddings share at least one edge. Thus, for a specific embedding *H*_*k*_, the degree of its corresponding node in $\bar {G^{o}}$ equals the number of embeddings overlapping with *H*_*k*_. Figure 3 depicts the overlap graph of the embeddings found in deterministic network *G*^*o*^ shown in Fig. 1. Consider a subset of embeddings $\mathcal {H}^{o} \subseteq \mathcal {H}(M|G^{o})$. We define an indicator function *ζ*() on $\mathcal {H}^{o}$ as follows: $\zeta (\mathcal {H}^{o}) = 1$ if no two embeddings in $\mathcal {H}^{o}$ share an edge, and $\zeta (\mathcal {H}^{o}) = 0$ otherwise.

**Fig. 4:**
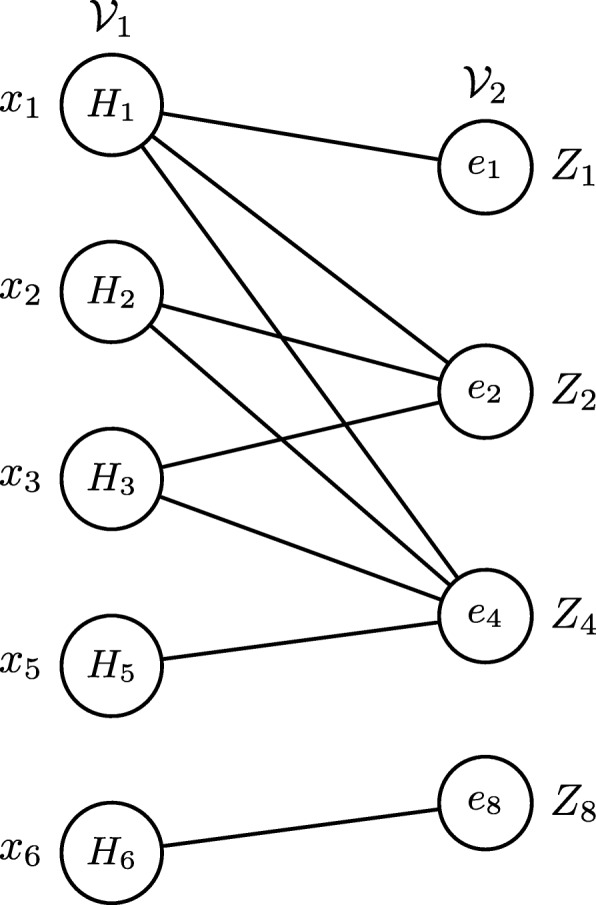
The bipartite graph *G*_4_ of the embedding *H*_4_. Each *x*_*i*_ denotes the variable for each node in $\mathcal {V}_{1}$. Each *Z*_*j*_ represents the edge polynomial for each node in $\mathcal {V}_{2}$

Consider a specific embedding *H*_*k*_ in *G*. Because of the uncertain nature of the probabilistic network, each embedding exists with a probability value. As a result, the number of embeddings overlapping with *H*_*k*_ is also uncertain. We represent it using a random variable *B*_*k*_. To calculate the distribution of *B*_*k*_, we construct a bipartite graph denoted with $G_{k} = (\mathcal {V}_{1}, \mathcal {V}_{2}, \mathcal {E})$. $\mathcal {V}_{1}$ and $\mathcal {V}_{2}$ represent two node sets, and $\mathcal {E}$ represents the edges connecting nodes of $\mathcal {V}_{1}$ with those of $\mathcal {V}_{2}$. Each neighboring node of *H*_*k*_ in the overlap graph corresponds to a node in $\mathcal {V}_{1}$. Each edge in the edge set, which constitutes all those overlapping embeddings of *H*_*k*_, corresponds to a node in $\mathcal {V}_{2}$. Notice that this edge set excludes the edges of embedding *H*_*k*_ itself. An edge exists between nodes $u \in \mathcal {V}_{1}$ and $v \in \mathcal {V}_{2}$ if the corresponding embedding of node *u* has the edge denoted by *v*. Figure 4 shows the bipartite graph *G*_4_ of embedding *H*_4_ in *G*^*o*^ (see Fig. 1). *H*_1_, *H*_2_, *H*_3_, *H*_5_ and *H*_6_ are neighbours of *H*_4_ in the overlap graph $\bar {G^{o}}$ (see Fig. 3). Thus these embeddings are nodes in $\mathcal {V}_{1}$ of *G*_4_. Their edges include *e*_1_,*e*_2_,*e*_3_,*e*_4_,*e*_5_,*e*_6_,*e*_7_ and *e*_8_. As edges *e*_3_, *e*_5_, *e*_6_ and *e*_7_ are also edges of *H*_4_, only *e*_1_,*e*_2_,*e*_4_ and *e*_8_ constitute $\mathcal {V}_{2}$ of *G*_4_.

To help better understand this paper, we introduce another two notations *x-polynomial* and *collapse operator*. Given a bipartite graph *G*_*k*_, we compute a polynomial, called the x-polynomial as follows. For each node $v_{i} \in \mathcal {V}_{1}$, it defines a unique variable *x*_*i*_. For each node $v_{j} \in \mathcal {V}_{2}$, the probability that *v*_*j*_’s corresponding edge is present and absent is *p*_*j*_ and *q*_*j*_ (*q*_*j*_=1−*p*_*j*_) respectively. For each node $v_{j} \in \mathcal {V}_{2}$, we construct a polynomial called *edge polynomial Z*_*j*_ as

1 $$  Z_{j}= p_{j} \prod\limits_{(v_{i},v_{j}) \in \mathcal{E}} x_{i} + q_{j}.  $$

The first term of this edge polynomial consists of the product of the variables of those overlapping embeddings containing this edge. The second term only has the probability of the absence of this edge. We explain the concept of edge polynomial using the example of the bipartite graph in Fig. 4. In this example, the edge polynomial for edge *e*_1_ is *Z*_1_=*p*_1_*x*_1_+*q*_1_. Also the edge polynomial corresponding to *e*_2_ is *Z*_2_=*p*_2_*x*_1_*x*_2_*x*_3_+*q*_2_. The first term of this edge polynomial represents the case that when edge *e*_2_ is present, it contributes to the existence of embeddings *H*_1_, *H*_2_ and *H*_3_ with a probability *p*_2_. The second term however represents the case that when edge *e*_2_ is absent with probability *q*_2_, none of those three embeddings exist. We compute the x-polynomial of *H*_*k*_ denoted with $\mathcal {Z}_{H_{k}}$ as

2 $$  \mathcal{Z}_{H_{k}} = \prod_{v_{j} \in \mathcal{V}_{2}} Z_{j}.  $$

The key characteristic of the x-polynomial in the above equation is that its terms model all possible deterministic network topologies for the edges denoted by $\mathcal {V}_{2}$. We write the *j*th term of the x-polynomial as $\alpha _{j} \prod _{v_{i} \in \mathcal {V}_{1}} x_{i}^{c_{ij}}$, where *α*_*j*_ is the probability and *c*_*ij*_ is the exponent of the variable *x*_*i*_. To compute this polynomial faster, we introduces a collapse operator for each variable *x*_*r*_ denoted with *ϕ*_*r*_(), as follows. Let us denote the degree of $v_{i} \in \mathcal {V}_{1}$ with *deg*(*v*_*i*_|*G*_*k*_). For each node’s unique variable *x*_*i*_, we define an indicator function *ψ*_*i*_(*c*), where *ψ*_*i*_(*c*)=1 if *c*=*deg*(*v*_*i*_|*G*_*k*_), otherwise *ψ*_*i*_(*c*)=0. Using these notations, for the *j*th term of the x-polynomial, we compute collapse operator *ϕ*_*r*_() as

3 $$ {}\phi_{r}\left(\alpha_{j} \prod_{v_{i} \in \mathcal{V}_{1}} x_{i}^{c_{ij}}\right) = [t \psi_{r}(c_{ij}) + (1 - \psi_{r}(c_{ij})] \alpha_{j} \prod_{v_{i} \in \mathcal{V}_{1} - \{v_{r}\}} x_{i}^{c_{ij}}.  $$

Notice that, the collapse operator *ϕ*_*r*_ only changes the variable *x*_*r*_. It either replaces it with *t* or completely removes it depending on the outcome of *ψ*_*r*_(). When *ψ*_*r*_()=1 (i.e., *c*_*rj*_=*deg*(*v*_*r*_|*G*_*k*_)), it means that all edges of embedding *H*_*r*_ are present (e.g., *H*_*r*_ exists). Thus, the variable *t* replaces *x*_*r*_ which means a motif is present. When *ψ*_*r*_()=0, it indicates that at least one edge of *H*_*r*_ is absent. Thus, the entire *H*_*r*_ is missing. For example, consider one of the terms resulting from the product of all edge polynomials in $\mathcal {Z}_{H_{4}}$, $q_{1}p_{2}p_{4}q_{8}x_{1}^{2}x_{2}^{2}x_{3}^{2}x_{5}$. If we apply the collapse operator *ϕ*_1_() to this term, the variable *x*_1_ will be removed as *ψ*_1_()=0 (*deg*(*H*_1_|*G*_4_) = 3 while the exponent of *x*_1_ in this term is 2). Similarly, if we apply the collapse operator *ϕ*_2_() to this term, the variable *x*_2_ will be replaced with *t* as *ψ*_2_()=1 (*deg*(*H*_2_|*G*_4_) = 2 and the exponent of *x*_2_ in this term is also 2). After applying all collapse operators to this term, it becomes *q*_1_*p*_2_*p*_4_*q*_8_*t*^3^ which indicates that when only edges *e*_2_ and *e*_4_ are present, there are three embeddings present. And this case happens with a probability *q*_1_*p*_2_*p*_4_*q*_8_. We apply the collapse operator *ψ*_*r*_ to the polynomial terms as soon as it completes multiplication of the final edge polynomial of the variable *x*_*r*_, which means that no other edge polynomial can increase the exponent of *x*_*r*_.

Given these definitions, we formally define two different independent motif counting problems next.

#### **Definition 1**

(INDEPENDENT MOTIF COUNTING IN PROBABILISTIC NETWORK I). Given a probabilistic network *G*=(*V*,*E*,*P*) and a motif pattern *M*, find a set of independent embeddings which yields the maximum expected number of occurrences in *G*, which is

4 $$\begin{array}{*{20}l}  {\underset{\substack{\mathcal{H}^{\prime}, \mathcal{H}^{\prime} \subseteq \mathcal{H}(M|G^{\prime}) \\ \zeta(\mathcal{H}^{\prime}) = 1}}{\arg\max}} \left\{ \sum_{G^{o} \in \mathcal{D}(G)} |\mathcal{H}(M|G^{o}) \cap \mathcal{H}^{\prime}| \cdot \mathcal{P}(G^{o}|G)\right\}. \end{array} $$

We explain the problem on a hypothetical probabilistic network *G*(see Fig. 2). To better explain the problem, we also list some possible deterministic networks in Fig. 2. Notice that this probabilistic network has the same network topology as the deterministic network *G*^*o*^ in Fig. 1a. As a result, *G* has six possible embeddings same with *G*^*o*^, which are *H*_1_, *H*_2_, *H*_3_, *H*_4_, *H*_5_ and *H*_6_ (see Fig. 1c-h). According to the problem definition, we seek to find a set of non-overlapping embeddings which contributes to the maximum expected number of motif count over all possible deterministic network topologies. For those six embeddings of *G*, we are able to construct five sets of independent embeddings, which are {*H*_1_,*H*_6_},{*H*_2_}, {*H*_3_}, {*H*_4_} and *H*_5_ (see Fig. 3 for the relationship between embeddings). For each set, we summarize the expected motif count over the set of all alternative deterministic network topologies based on Eq. 4. Table 1 lists the result. Then, we choose the set with maximum motif count. Notice that, the resulting embedding set with the maximum expected motif count is not guaranteed to always have the largest motif frequency among all possible deterministic networks. For example, in deterministic network $G_{1}^{o}$, the set {*H*_1_,*H*_6_} has the highest motif frequency; while in network $G_{3}^{o}$, it is the set {*H*_2_} achieves the largest motif count. By requiring to select the set of embeddings with highest frequency in each possible deterministic network, we have our second independent motif counting problem. We formally define it next.

**Table 1:** {*H*_1_,*H*_6_}, {*H*_2_}, {*H*_3_}, {*H*_4_} and {*H*_5_} are the five possible independent embedding sets of the motif *M* (Fig. 1) in network *G* (Fig. 2). The table shows the number of embeddings occurring at each deterministic network for each independent embedding set and its expected value in *G*

	$G_{1}^{o}$	$G_{2}^{o}$	$G_{3}^{o}$	…	Expected motif count
{*H*_1_,*H*_6_}	2	1	0	…	$2\times \mathcal {P}(G_{1}^{o}|G)+1\times \mathcal {P}(G_{2}^{o}|G)+0\times \mathcal {P}(G_{3}^{o}|G)+\dots $
{*H*_2_}	1	1	1	…	$1\times \mathcal {P}(G_{1}^{o}|G)+1\times \mathcal {P}(G_{2}^{o}|G)+1\times \mathcal {P}(G_{3}^{o}|G)+\dots $
{*H*_3_}	1	1	0	…	$1\times \mathcal {P}(G_{1}^{o}|G)+1\times \mathcal {P}(G_{2}^{o}|G)+0\times \mathcal {P}(G_{3}^{o}|G)+\dots $
{*H*_4_}	1	1	0	…	$1\times \mathcal {P}(G_{1}^{o}|G)+1\times \mathcal {P}(G_{2}^{o}|G)+0\times \mathcal {P}(G_{3}^{o}|G)+\dots $
{*H*_5_}	1	1	0	…	$1\times \mathcal {P}(G_{1}^{o}|G)+1\times \mathcal {P}(G_{2}^{o}|G)+0\times \mathcal {P}(G_{3}^{o}|G)+\dots $

#### **Definition 2**

(INDEPENDENT MOTIF COUNTING IN PROBABILISTIC NETWORK II). Given a probabilistic network *G*=(*V*,*E*,*P*) and a motif pattern *M*, compute the expected number of maximum independent occurrences of *M* in *G*, which is

5 $$\begin{array}{*{20}l}  \sum\limits_{G^{o} \in \mathcal{D}(G)} {\underset{\substack{\mathcal{H}^{o}, \mathcal{H}^{o} \subseteq \mathcal{H}(M|G^{o}) \\ \zeta(\mathcal{H}^{o}) = 1}}{\arg\max}} {|\mathcal{H}^{o}|} \cdot \mathcal{P}(G^{o}|G). \end{array} $$

Notice that in this problem, we are required to always select the largest independent embedding set in each possible deterministic network topology. We compute the expected number of independent motif by iterating over all possible deterministic networks and summing up the motif count. For example, in the example network (Fig. 2), the expected independent motif count is calculated by $2 \cdot \mathcal {P}(G_{1}^{o}|G) + 1 \cdot \mathcal {P}(G_{2}^{o}|G) + 1 \cdot \mathcal {P}(G_{3}^{o}|G) + \dots $.

The former definition of the independent motif counting problem above (Definition 1) seeks the genes, which are more likely to carry out the function characterized by the given motif across all possible deterministic topologies. The latter definition (Definition 2) does not care about the identity of the set of genes engaged in the process as the set of genes vary depending on the deterministic network topology observed. It instead counts the number of different ways we can observe the process separately for each topology even though that set may differ from one topology to another. In this paper, we focus on the first problem. The rationale is that we often do not know the specific deterministic topology realized at a given point in time. Furthermore, this topology can vary over time. Notice that this problem can be solved by enumerating all possible deterministic network topologies and independent embedding sets. However, it is infeasible to scale to large networks as the numbers of deterministic network topologies and independent embedding sets grow exponentially. In this paper, we develop a scalable method to tackle this problem by utilizing a polynomial model and three strategies. We discuss this polynomial model and three strategies next.

### Overview of the existing solution

Here, we briefly describe the method by Sarkar et al. [24] for counting independent motif instances, as our method utilizes the same polynomial model in that study. Given a probabilistic graph *G*=(*V,E,P*) and the specified motif pattern *M*, the algorithm works in three steps. First, it discovers all motif embeddings in the deterministic network *G*^′^=(*V*,*E*). It then builds an overlap graph for these embeddings. Next, it uses a heuristic strategy to count non-overlapping motif embeddings; it calculates a priority value for each node (we explain how to compute priority value below) and iteratively picks the node with the highest priority in the overlap graph. It includes the corresponding embedding to the result set, adds the probability that this embedding exists to the motif count and removes this node along with all of its neighbouring nodes from the overlap graph. It repeats this process until the graph is empty.

The key step of this method is calculating the priority value for each node in the overlap graph. The priority value of a node primarily depends on the number of neighbours of a node. In a probabilistic networks, both the existences of an embedding and its overlapping embeddings are uncertain as the edges which make up those embeddings are probabilistic. To accurately model this uncertainty, for each embedding *H*_*k*_, it first calculates a gain value *a*_*k*_, which equals to the probability that *H*_*k*_ exists $\left (a_{k} = \prod _{e \in H_{k}} P(e)\right)$. Then it computes a loss value using the number of neighbours of *H*_*k*_ which is represented with a random variable *B*_*k*_. It then computes the loss value of *H*_*k*_ as a function of *B*_*k*_, denoted with *f*(*B*_*k*_). Finally, it determines the priority value, denoted with *ρ*_*k*_, as a function of gain value and loss value. In this paper, we compute *ρ*_*k*_ as *a*_*k*_/*f*(*B*_*k*_).

Sarkar et al. compute the distribution of *B*_*k*_ using a x-polynomial. To construct this x-polynomial, it first builds an undirected bipartite graph denoted with $G_{k} = (\mathcal {V}_{1},\mathcal {V}_{2}, \mathcal {E})$. Then for each node $v_{j} \in \mathcal {V}_{2}$, it constructs an edge polynomial *Z*_*j*_. After multiplying all edge polynomials and collapsing it, the x-polynomial takes the form

6 $$  \mathcal{Z}_{H_{k}} = \sum\limits_{j = 0}^{s} p_{kj} t^{j}.  $$

The coefficients of the polynomial $\mathcal {Z}_{H_{k}}$ is the true distribution of the random variable *B*_*k*_ (i.e., ∀*j*, the coefficient of *t*^*j*^ is the probability that *B*_*k*_=*j*). For any further information, we refer the interested readers to [24].

### Avoiding loss computation

Recall that, we calculate the distribution of *B*_*k*_ for all nodes of the overlap graph only to select the one that yields the highest priority value *ρ*_*k*_() (see “Overview of the existing solution section”). Here, we develop a method to quickly compute an upper bound to *ρ*_*k*_. This allows us to avoid computation of the distribution of *B*_*k*_ for the node *v*_*k*_ when the upper bound to *ρ*_*k*_ is less than *ρ*_*j*_ for any node *v*_*j*_ considered prior to *v*_*k*_. To explain this strategy, we first present our theory which establishes the foundation of the upper bound computation. We start by defining our notation.

Consider $G_{k} = (\mathcal {V}_{1}, \mathcal {V}_{2}, \mathcal {E})$ of an embedding *H*_*k*_. For a given subset $\mathcal {V}_{2}^{\prime } \subseteq \mathcal {V}_{2}$, let us denote the x-polynomial of *H*_*k*_ after multiplying the edge polynomials of node set $\mathcal {V}_{2}^{\prime }$ with $Z_{H_{k},\mathcal {V}_{2}^{\prime }}$. Below, we discuss our theory using a lemma, a theorem, and a corollary.

#### **Lemma 1**

Consider the bipartite graph of motif embedding *H*_*k*_ denoted with ${G_{k}=(\mathcal {V}_{1},\mathcal {V}_{2}, \mathcal {E})}$. For all nodes $v_{r} \in \mathcal {V}_{2}-\mathcal {V}_{2}^{\prime }$, $\forall \tau \in \{0,1,2,\dots,|\mathcal {V}_{1}|\}$, we have

$$P\left(B_{k} \ge \tau | Z_{H_{k},\mathcal{V}_{2}^{\prime}}\right) \le P\left(B_{k} \ge \tau | Z_{H_{k},\mathcal{V}_{2}^{\prime} \cup {v_{r}}}\right). $$

#### *Proof*

We expand $P\left (B_{k} \ge \tau | Z_{H_{k},\mathcal {V}_{2}^{\prime }}\right)$ as

7 $$  P\left(B_{k} \ge \tau | Z_{H_{k},\mathcal{V}_{2}^{\prime}}\right) = \sum\limits_{\tau^{\prime} = \tau}^{|\mathcal{V}_{1}|} P\left(B_{k}=\tau^{\prime} | Z_{H_{k},\mathcal{V}_{2}^{\prime}}\right).  $$

We first discuss how to compute the probability that exactly *τ*^′^ neighboring embeddings of *H*_*k*_ exist. After multiplying edge polynomials and collapsing, $Z_{H_{k},\mathcal {V}_{2}^{\prime }}$ takes the following form:

$$\begin{array}{*{20}l} Z_{H_{k},\mathcal{V}_{2}^{\prime}} =& \phi_{1} \left(\phi_{2} \left(\dots \phi_{|\mathcal{V}_{1}|} \left(\prod_{\substack{v_{j} \in \mathcal{V}_{2}^{\prime} \\ \mathcal{V}_{2}^{\prime} \subset \mathcal{V}_{2}}} Z_{j}\right)\right) \right) \\ =& \sum\limits_{j} t^{j}\left(\sum\limits_{l} \left(\alpha_{jl} \prod\limits_{v_{i} \in \mathcal{V}_{1}}x_{i}^{c_{ijl}}\right)\right). \end{array} $$

Here, ${\sum \nolimits }_{l} \alpha _{jl}$, which sums up all the coefficients of the polynomial terms containing *t*^*j*^, equals to the probability that exactly *j* neighboring embeddings of *H*_*k*_ exist after multiplying the edge polynomials of $\mathcal {V}_{2}^{\prime }$. Next, we focus on one polynomial term from the above x-polynomial. Let us denote this polynomial term as $ A = \alpha t^{j} \prod \limits _{v_{i} \in \mathcal {V}_{1}}x_{i}^{c_{i}} $. Let us define an indicator function *δ*_*r*_(*i*), where *δ*_*r*_(*i*)=1 if $(v_{i},v_{r}) \in \mathcal {E}$, otherwise *δ*_*r*_(*i*)=0. Then after multiplying one more edge polynomial, say $Z_{r} = p_{r} \prod \limits _{(v_{i},v_{j}) \in \mathcal {E}} x_{i} + (1-p_{r})$, the polynomial term *A* expands into two polynomial terms denoted as *B*+*C*, where $B=p_{r} \alpha t^{j} \prod \limits _{v_{i} \in \mathcal {V}_{1}}x_{i}^{c_{i}+\delta _{r} (i)}$ and $C=(1-p_{r}) \alpha t^{j} \prod \limits _{v_{i} \in \mathcal {V}_{1}}x_{i}^{c_{i}}$. Two cases may happen after the collapsing of the polynomial terms *B* and *C*.

*Case 1: There is no collapse.* The exponent of the variable *t* of polynomial terms *B* and *C* remains the same. Adding up the coefficients of term *t*^*j*^, we get

$$\alpha p_{r} + \alpha (1-p_{r}) = \alpha.$$

Thus, after multiplying another edge polynomial, the coefficient of term *t*_*j*_ remains the same. In other words, multiplying another edge polynomial has no effect on *P*(*B*_*k*_≥*τ*). Mathematically, $P\left (B_{k} \ge \tau | Z_{H_{k},\mathcal {V}_{2}^{\prime }}\right) = P\left (B_{k} \ge \tau | Z_{H_{k},\mathcal {V}_{2}^{\prime } \cup {v_{r}}}\right)$.

*Case 2: There is collapse.* In this case, the exponent of the variable *t* of polynomial term *B* will increase while it stays the same for polynomial term *C*, since multiplying the second term of *Z*_*r*_ does not introduce any *x* variable. Let us denote the increment in the exponent of *t* (i.e., the number of *x*_*i*_ variables which collapse after multiplying *Z*_*r*_) with *j*_0_. Now the polynomial terms *B* and *C* become $p_{r} \alpha t^{j+j_{0}} \prod \limits _{v_{i} \in \mathcal {V}_{1}}x_{i}^{c_{i}}$ and $(1-p_{r}) \alpha t^{j} \prod \limits _{v_{i} \in \mathcal {V}_{1}}x_{i}^{c_{i}}$ respectively. How this multiplication affects *P*(*B*_*k*_≥*τ*) depends on the relationship between *j* and *τ*. We have two cases:

*Case 2.a* When *j*<*τ*, polynomial term *A* does not contribute to *P*(*B*_*k*_≥*τ*) before multiplying *Z*_*r*_. After multiplying *Z*_*r*_, polynomial term *C* also does not contribute to *P*(*B*_*k*_≥*τ*). Whether polynomial term *B* contributes to *P*(*B*_*k*_≥*τ*) depends on the relationship between *j*+*j*_0_ and *τ*. If *j*+*j*_0_≥*τ*, the probability that *j*+*j*_0_ neighboring embeddings of *H*_*k*_ exist grows. Thus, based on the Eq. 7, *P*(*B*_*k*_≥*τ*) increases by *p*_*r*_*α* (i.e., the coefficient of $\phantom {\dot {i}\!}t^{(j+j_{0})}$). On the other hand, if *j*+*j*_0_<*τ*, polynomial term *B* has no effect on *P*(*B*_*k*_≥*τ*). In conclusion, after multiplying one more edge polynomial, the value of *P*(*B*_*k*_≥*τ*) either increases or remains the same. Mathematically, $P\left (B_{k} \ge \tau | Z_{H_{k},\mathcal {V}_{2}^{\prime }}\right) \le P\left (B_{k} \ge \tau | Z_{H_{k},\mathcal {V}_{2}^{\prime } \cup {v_{r}}}\right)$.

*Case 2.b* When *j*≥*τ*, the polynomial term *A* contributes to *P*(*B*_*k*_≥*τ*). From Eq. 7, before multiplying *Z*_*r*_, the amount of contribution of polynomial term *A* to *P*(*B*_*k*_≥*τ*) is *α*. After multiplying *Z*_*r*_, the amount of contribution is equal to the sum of the coefficients of the polynomial terms *B* and *C*, where is *αp*_*r*_+*α*(1−*p*_*r*_)=*α*. Thus, *P*(*B*_*k*_≥*τ*) remains the same. Mathematically, $P\left (B_{k} \ge \tau | Z_{H_{k},\mathcal {V}_{2}^{\prime }}\right) = P\left (B_{k} \ge \tau | Z_{H_{k},\mathcal {V}_{2}^{\prime } \cup {v_{r}}}\right)$. □

The above lemma leads to the following theorem:

#### **Theorem 1**

Consider a motif embedding *H*_*k*_ and its corresponding bipartite graph $G_{k} = (\mathcal {V}_{1}, \mathcal {V}_{2}, \mathcal {E})$. Also consider a subset $\mathcal {V}_{2}^ \prime \subset \mathcal {V}_{2}$. Given a monotonic function $\gamma (): \mathbb {R} \to \mathbb {R}$ such that *γ*(0)=0 and for ∀*x*≥*y*≥0, *γ*(*x*)≥*γ*(*y*)≥0. $\forall v_{r} \in \mathcal {V}_{2}-\mathcal {V}_{2}^{\prime }$, we have

$${}\sum\limits_{j=0}^{|\mathcal{V}_{1}|} \gamma (j) P\left(B_{k} = \!j | Z_{H_{k},\mathcal{V}_{2}^ \prime}\right) \le \sum\limits_{j=0}^{|\mathcal{V}_{1}|} \gamma (j) P\left(B_{k} = j | Z_{H_{k},\mathcal{V}_{2}^ \prime \cup v_{r}}\right).$$

#### *Proof*

From the monotonicity of *γ*() function, for ∀*j*≥1, we have

$$\gamma (j) - \gamma (j-1) \ge 0.$$

From Lemma 1, given $\mathcal {V}_{2}^ \prime $ and $v_{r} \in \mathcal {V}_{2} - \mathcal {V}_{2}^ \prime $, for ∀*j*≥0, we have

$$P\left(B_{k} \ge j | Z_{H_{k}, \mathcal{V}_{2}^ \prime}\right) \le P\left(B_{k} \ge j | Z_{H_{k}, \mathcal{V}_{2}^ \prime \cup v_{r}}\right).$$

For ∀*j*≥1, by multiplying both sides of the inequality with (*γ*(*j*)−*γ*(*j*−1)), we get

$$\begin{aligned} &(\gamma (j) - \gamma (j-1)) P\left(B_{k} \ge j | Z_{H_{k}, \mathcal{V}_{2}^ \prime}\right)\\ &\quad\le (\gamma (j) - \gamma (j-1)) P(B_{k} \ge j | Z_{H_{k}, \mathcal{V}_{2}^ \prime \cup v_{r}}). \end{aligned} $$

Thus, summing up this inequality $\forall j \le |\mathcal {V}_{1}|$, we get

8 $$ \begin{aligned} &\sum\limits_{j=1}^{|\mathcal{V}_{1}|} (\gamma (j) - \gamma (j-1)) P\left(B_{k} \ge j | Z_{H_{k}, \mathcal{V}_{2}^ \prime}\right)\\ &\quad \le \sum\limits_{j=1}^{|\mathcal{V}_{1}|} (\gamma (j) - \gamma (j-1)) P\left(B_{k} \ge j | Z_{H_{k}, \mathcal{V}_{2}^ \prime \cup v_{r}}\right). \end{aligned}  $$

We rewrite the left side of this inequality as

9 $$ {{\begin{aligned} & \sum\limits_{j=1}^{|\mathcal{V}_{1}|} (\gamma (j) - \gamma (j-1)) P(B_{k} \ge j | Z_{H_{k}, \mathcal{V}_{2}^ \prime}) \\ &= \sum\limits_{j=1}^{|\mathcal{V}_{1}|} \gamma (j) P\left(B_{k} \ge j | Z_{H_{k}, \mathcal{V}_{2}^ \prime}\right) - \sum\limits_{j=1}^{|\mathcal{V}_{1}|} \gamma (j-1) P\left(B_{k} \ge j | Z_{H_{k}, \mathcal{V}_{2}^ \prime}\right) \\ &= \sum\limits_{j=1}^{|\mathcal{V}_{1}|} \gamma (j) P\left(B_{k} \ge j | Z_{H_{k}, \mathcal{V}_{2}^ \prime}\right) - \sum\limits_{j=0}^{|\mathcal{V}_{1}|-1} \gamma (j) P\left(B_{k} \ge j+1 | Z_{H_{k}, \mathcal{V}_{2}^ \prime}\right) \\ &= \sum\limits_{j=1}^{|\mathcal{V}_{1}|-1} {\gamma (j) \left[ P\left(B_{k} \ge j | Z_{H_{k}, \mathcal{V}_{2}^ \prime}\right) - P\left(B_{k} \ge j+1 | Z_{H_{k}, \mathcal{V}_{2}^ \prime}\right) \right]} \\ &{}+ \gamma ({|\mathcal{V}_{1}|}) P\left(B_{k} \ge {|\mathcal{V}_{1}|} | Z_{H_{k}, \mathcal{V}_{2}^ \prime}\right) - \gamma (0)P\left(B_{k} \ge 1 | Z_{H_{k}, \mathcal{V}_{2}^ \prime}\right) \end{aligned}}}  $$

Given *P*(*B*_*k*_=*j*)=*P*(*B*_*k*_≥*j*)−*P*(*B*_*k*_≥*j*+1) and *γ*(0)=0, we rewrite Eq. 9 as

$$\begin{array}{*{20}l} &{} \sum\limits_{j=1}^{|\mathcal{V}_{1}|} (\gamma (j) - \gamma (j-1)) P(B_{k} \ge j | Z_{H_{k}, \mathcal{V}_{2}^ \prime}) \\ &{}= \sum\limits_{j=1}^{|\mathcal{V}_{1}|-1}\! {\gamma (j) P\left(B_{k} \,=\, j | Z_{H_{k}, \mathcal{V}_{2}^ \prime}\!\right)} \,+\, \gamma (|\mathcal{V}_{1}|) P\!\left(B_{k} \!= {\!|\mathcal{V}_{1}| | Z_{H_{k}, \mathcal{V}_{2}^ \prime}}\!\right) \\ &{}= \sum\limits_{j=1}^{|\mathcal{V}_{1}|} {\gamma (j) P\left(B_{k} = j | Z_{H_{k}, \mathcal{V}_{2}^ \prime}\right)} \end{array} $$

Similarly, we rewrite the right side of Inequality (8) as

$$\begin{aligned} \sum\limits_{j=1}^{|\mathcal{V}_{1}|} (\gamma (j) - \gamma (j-1)) P\left(B_{k} \ge j | Z_{H_{k}, \mathcal{V}_{2}^ \prime \cup v_{r}}\right)\\[-12pt] =\sum\limits_{j=1}^{|\mathcal{V}_{1}|} {\gamma (j) P\left(B_{k} = j | Z_{H_{k}, \mathcal{V}_{2}^ \prime \cup v_{r}}\right)}. \end{aligned} $$

Using the above equations, We rewrite the Inequality (8) as

$${}\sum\limits_{j=1}^{|\mathcal{V}_{1}|} \gamma (j) P\left(B_{k} = j | Z_{H_{k},\mathcal{V}_{2}^ \prime}\right) \le \sum\limits_{j=1}^{|\mathcal{V}_{1}|} \gamma (j) P\left(B_{k} \,=\, j | Z_{H_{k},\mathcal{V}_{2}^ \prime \cup v_{r}}\right). $$

As *γ*(0)=0, using the above inequality, we get

$${}\sum\limits_{j=0}^{|\mathcal{V}_{1}|} \gamma (j) P\left(B_{k} = j | Z_{H_{k},\mathcal{V}_{2}^ \prime}\right) \le \sum\limits_{j=0}^{|\mathcal{V}_{1}|} \gamma (j) P\left(B_{k} \,=\, j | Z_{H_{k},\mathcal{V}_{2}^ \prime \cup v_{r}}\right). $$

□

This theorem gives us a general form of *f*(*B*_*k*_) function which is monotonically increasing. For example, the expected value of *B*_*k*_, *Exp*(*B*_*k*_) falls into that category. Corollary below proves it:

#### **Corollary 1**

Given $\mathcal {V}_{2}^ \prime $ and $v_{r} \in \mathcal {V}_{2} - \mathcal {V}_{2}^ \prime $, the expected number of neighboring embeddings of *H*_*k*_ monotonically increases with growing edge polynomial set:

$$Exp(B_{k} | Z_{H_{k},\mathcal{V}_{2}^{\prime}}) \le Exp\left(B_{k} | Z_{H_{k},\mathcal{V}_{2}^{\prime} \cup {v_{r}}}\right).$$

#### *Proof*

The expected value of *B*_*k*_ can be computed as

$$Exp(B_{k}) = \sum\limits_{j=0}^{|\mathcal{V}_{1}|} {jP(B_{k}=j)}.$$

We have *γ*(*j*)=*j* which is a monotonical function. Thus, from Theorem 1, we have

$$Exp\left(B_{k} | Z_{H_{k},\mathcal{V}_{2}^{\prime}}\right) \le Exp\left(B_{k} | Z_{H_{k},\mathcal{V}_{2}^{\prime} \cup {v_{r}}}\right). $$

□

Using Theorem 1, we develop our method for avoiding the costly computation of the distribution of *B*_*k*_ for each embedding *H*_*k*_ of the given motif in the target network. Our method works for all monotonic loss functions (e.g., *f*(*B*_*k*_)=*Exp*(*B*_*k*_)). Assume that, ∃*k*>1, ∀*i*1≤*i*<*k*, we already computed the values *a*_*i*_, distribution of *B*_*i*_, *f*(*B*_*i*_), and thus *ρ*_*i*_. Let us denote the largest observed priority value so far with *ρ*^⋆^=*max*_1≤*i*<*k*_{*ρ*_*i*_}. We explain next how we use this information to avoid computation of the distribution of *B*_*k*_ whenever possible. Let us denote the bipartite graph of *H*_*k*_ with $G_{k} = (\mathcal {V}_{1}, \mathcal {V}_{2}, \mathcal {E})$. Our algorithm iteratively multiplies the edge polynomials for all the nodes in $\mathcal {V}_{2}$ one by one and collapses the resulting polynomial. Let us denote the set of nodes in *V*_2_ with $\mathcal {V}_{2} = v_{1}, v_{2}, \dots, v_{|\mathcal {V}_{2}|}$. Without losing generality, let us assume that we multiply the edge polynomials in the order $v_{1}, v_{2}, \dots, v_{|\mathcal {V}_{2}|}$. ∀*j*, $1 \le j \le |\mathcal {V}_{2}|$, after multiplying the first *j* polynomials, we get an intermediate probability distribution $B_{k}^ j$. Using that distribution $B_{k}^ j$, we compute an intermediate priority value for *H*_*k*_ and denote it with $\rho _{k}^ j$. Recall that Theorem 1 states that ∀*j*, $\rho _{k}^{j} \le \rho _{k}^{j-1}$ (i.e., the priority value monotonically decreases if loss value monotonically increases). Following from this theorem, we terminate computation of *B*_*k*_ as soon as $\rho _{k}^ j$ becomes less than the best priority value observed, *ρ*^⋆^. This eliminates costly polynomial multiplication for *H*_*k*_.

When to stop the calculation of *B*_*k*_ largely depends on the best priority value *ρ*^⋆^ observed so far. The larger *ρ*^⋆^ is, the sooner we terminate the computation of *B*_*k*_. Thus, the ideal ordering places embeddings with larger priority values should earlier. The dilemma here is that we do not know the priority values of the embeddings at this stage. Therefore, we use a proxy value of each embedding *H*_*k*_, denoted with *Q*_*k*_, which is trivial to compute, *a*_*k*_ (i.e., the gain value of *H*_*k*_) divided by the number of overlapping embeddings with *H*_*k*_. We rank embeddings in descending order of their *Q*_*k*_ values. The rationale behind using the value *Q*_*k*_ is as follows. Recall that the priority value of *H*_*k*_ is determined by the gain value *a*_*k*_ and the loss value, which largely depends on the distribution of its neighboring nodes. *Q*_*k*_ conjectures that the larger the degree of the corresponding node of *H*_*k*_ is, the larger its loss value is. Thus, *Q*_*k*_ is inversely proportional to the number of embeddings, which conflict with *H*_*k*_.

### Efficient polynomial collapsation

Collapsation plays an important role in calculating the distribution of *B*_*k*_ of the embedding *H*_*k*_ efficiently. The sooner we collapse the polynomial terms, the earlier we compute an upper bound to *ρ*_*k*_. Here, we introduce two orthogonal strategies to ensure early collapsation during the construction of the x-polynomial. We describe our strategies on the bipartite graph $G_{k} = (\mathcal {V}_{1}, \mathcal {V}_{2}, \mathcal {E})$ of *H*_*k*_. Our first strategy focuses on $\mathcal {V}_{1}$. The second one focuses on $\mathcal {V}_{2}$.

**Optimization on** $\mathcal {V}_{1}$ In order to collapse an x-polynomial term, which contains variable *x*_*i*_, we need to multiply all the edge polynomials containing *x*_*i*_ (see Eq. 3). The degree of the node $v_{i} \in \mathcal {V}_{1}$, *deg*(*v*_*i*_|*G*_*k*_) is equal to the number of edge polynomials that the variable *x*_*i*_ needs to be collapsed. Consider a node $v_{i} \in \mathcal {V}_{1}$ with *deg*(*v*_*i*_|*G*_*k*_)=1. Suppose that $ \exists v_{j} \in \mathcal {V}_{2}$, $(v_{i},v_{j}) \in \mathcal {E}$. We collapse the variable *x*_*i*_ as soon as the edge polynomial *Z*_*j*_ has been multiplied into the x-polynomial. In this case, the collapse operator *ϕ*_*i*_ will replace the variable *x*_*i*_ in x-polynomial with *t*. Following from this observation, our first strategy works as follows. Consider a node $v_{j} \in \mathcal {V}_{2}$. Let us denote the set of all nodes $v_{i} \in \mathcal {V}_{1}$ for which *deg*(*v*_*i*_|*G*_*k*_)=1 and $(v_{i},v_{j}) \in \mathcal {E}$ with $\mathcal {V}_{1,j}$. We rewrite Eq. 1 (see Section “Preliminaries and problem definition”) as

$$Z_{j}= p_{j} t^{|\mathcal{V}_{1,j}|} \prod_{\substack {v_{i} \in \mathcal{V}_{1} - \mathcal{V}_{1,j} \\ (v_{i},v_{j}) \in \mathcal{E}}} x_{i} + q_{j}. $$

The above equation means that before we do any polynomial multiplication, we first apply the collapse operator *ϕ*_*i*_ (∀*i*, such that $v_{i} \in \mathcal {V}_{1,j}$) to *Z*_*j*_. This preemptive collapsation prevents the exponential growth in the number of collapsation operations for those *v*_*i*_ satisfying the conditions above. For example, in the example bipartite graph (see Fig. 4), for edge *e*_8_, its original edge polynomial *Z*_8_=*p*_8_*x*_6_+*q*_8_ can be rewritten as *Z*_8_=*p*_8_*t*+*q*_8_ to avoid applying the collapse operation *ϕ*_6_() in any further polynomial multiplication.

**Optimization on** $\mathcal {V}_{2}$ The order in which edge polynomials are multiplied has a great effect on the cost of polynomial multiplication. Recall from “Preliminaries and problem definition section” that each variable *x*_*r*_ collapses only after the multiplication of the final edge polynomial for *x*_*r*_ has been completed. Following from this observation, our second strategy conjectures that increasing the number of collapsing variables after the product of a given number of edge polynomials reduces both the running time and the amount of memory needed to store the x-polynomial. We explain this on the bipartite graph in Fig. 4. In our example, to simplify our notation, we will only consider the optimization on $\mathcal {V}_{2}$ and ignore the impact of the optimization on $\mathcal {V}_{1}$ described above. We have four edge polynomials in total. If we multiply four edge polynomials in the order of *Z*_1_,*Z*_2_,*Z*_4_,*Z*_8_, no collapsation takes place until we multiply *Z*_4_. This is because *Z*_4_ is the final edge polynomial for *x*_1_, *x*_2_, *x*_3_ and *x*_5_. As a result, this ordering requires in total 32 collapses (i.e., number of polynomial terms is 2^3^= 8, and we collapse each of *x*_1_, *x*_2_, *x*_3_ and *x*_5_ resulting in 8+8+8+8 operations). Adding the collapsation cost of the last edge polynomial, 2^4^=16, we achieve 32 + 16 = 48 operations in total. Now, let us analyse the cost of the same product when we multiply the edge polynomials in the order of (*Z*_4_,*Z*_2_,*Z*_8_,*Z*_1_). In this ordering, *Z*_4_ is the final edge polynomial for *x*_5_. Thus, we need another 2^1^ = 2 operations for variable *x*_5_. The following edge polynomial *Z*_2_ is the final edge polynomial for variables *x*_2_ and *x*_3_. Therefore, once we multiply *Z*_2_, we can collapse variables *x*_2_ and *x*_3_. Thus, for variables *x*_2_ and *x*_3_, only 8 collapses are needed (i.e., there are 4 polynomial terms and 2 collapse operators). Variables *x*_6_ and *x*_1_ collapses after the product of *Z*_8_ and *Z*_1_ leading to eight and sixteen more collapse operations respectively. In total, this ordering yields only 34 (i.e., 2+8+8+16) collapses. By reordering the edge polynomials, we reduce not only the time for collapsation, but also the memory space for storing the variables. Furthermore, as we explain below, an effective ordering has potential to avoid the loss computation without losing the accuracy of the result. Next, we formally define the problem of ordering of the edge polynomials.

#### **Definition 3**

ORDERING OF THE EDGE POLYNOMIALS. Assume a bipartite graph $G_{k} = (\mathcal {V}_{1}, \mathcal {V}_{2}, \mathcal {E})$ and a specified loss value denoted by *ε* are given. Each node $v_{i} \in \mathcal {V}_{1}$ has a unique variable *x*_*i*_ and a collapse operator *ϕ*_*i*_. Each node $v_{j} \in \mathcal {V}_{2}$ has an edge polynomial *Z*_*j*_. For each collapse operator *ϕ*_*i*_, let us denote the number of the polynomial terms it has been applied to with $N_{\phi _{i}}$. Let us denote a permutation of the integers in the $[1:|\mathcal {V}_{2}|]$ interval with *π*=[*π*_1_, *π*_2_, …, $\pi _{|\mathcal {V}_{2}|}]$. Our problem is to find the ordering *π*, for which ∃*r*, such that after multiplying the first *r* polynomials in the order of *π*, we have *f*(*B*_*k*_)≥*ε* and $r|\mathcal {V}_{1}|2^{r} + \sum _{s=1}^{|\mathcal {V}_{1}|}N_{\phi _{s}}$ is minimized.

Notice that in the definition above, we aim to minimize the number of edge polynomials (i.e., variable *r*). Also if there are multiple orderings with the same number of edge polynomials, we prefer the one that requires the least collapsation operations (i.e., ${\sum \nolimits }_{s=1}^{|\mathcal {V}_{1}|}N_{\phi _{s}}$).

One straightforward method to solve this problem is to calculate the collapsation cost for all possible orderings of the edge polynomials and choose the one with the smallest cost. This, however, is infeasible as there are $|\mathcal {V}_{2}|$! alternative orderings. Here, we develop a greedy iterative algorithm to quickly estimate an ordering. Briefly, at each iteration, our algorithm chooses the edge which contributes to the collapsation of most variables. We explain our algorithm using the bipartite graph shown in Fig. 4.

Our algorithm maintains two matrices. We denote these two matrices at the *i*th iteration with *W*_*i*_ and *D*_*i*_. At each stage, we update the *W*_*i*_ matrix and generate a *D*_*i*_ matrix based on *W*_*i*_. We will explain these two matrices in detail later. We choose a suitable edge polynomial using the *D*_*i*_ matrix, and repeat this process until the last edge polynomial.

**Fig. 5:**
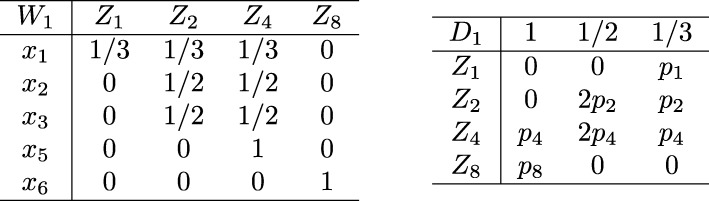
The two matrices *W*_1_ and *D*_1_ we maintained to order the edge polynomials

We first explain the two matrices above. The first matrix denoted by *W*_*i*_ maintains the relationship between the *x* variables and edge polynomials. Let us denote the *r*th row and *s*th column of *W*_*i*_ with *W*_*i*_[*r*,*s*]. Also, let us denote the bipartite graph of *H*_*k*_ at the *i*th iteration with $G_{k}^{i}$. If the x-polynomial *Z*_*s*_ contains the variable *x*_*r*_, we set $W_{i}[r, s] = 1/deg\left (v_{r}| G_{k}^{i}\right)$. Otherwise, we set *W*_*i*_[*r*,*s*]=0. Conceptually, this number indicates the contribution of the edge polynomial *Z*_*s*_ to collapse variable *x*_*r*_. For instance, *W*_*i*_[*r*,*s*]=1 implies that *Z*_*s*_ is the final edge polynomial of *x*_*r*_. Figure 5 presents matrix *W*_1_ corresponding to the bipartite graph in Fig. 4. Here, *Z*_2_ and *Z*_4_ contain *x*_2_. As a result *W*_1_[2,2]=*W*_1_[2,3]=1/2.

We construct matrix *D*_*i*_ from *W*_*i*_. This matrix counts different levels of contributions of each edge polynomial. It has $|\mathcal {V}_{2}|$ rows and $\max _{u \in \mathcal {V}_{1}}\{deg(u | G_{k}^{i})\}$ columns. We set the entry *D*_*i*_[*r*,*s*] to the product of the number of entries in the *r*th column of *W*_*i*_ having the value 1/*s* and the edge probability in *Z*_*r*_. For example, in the matrix *W*_1_ in Fig. 5, *Z*_2_ (i.e., column 2) contributes two variables at value 1/2 and one variable 1/3. As a result, we set the second row of *D*_1_ to [0, 2 *p*_2_, *p*_2_].

At the *i*th iteration, using the matrix *D*_*i*_, we choose the next edge polynomial for multiplication as follows. We start by looking at the first column of *D*_*i*_, and choose the row (i.e., edge polynomial) with the largest value. If there are multiple such rows, we use the second column of *D*_*i*_ among those polynomials. We repeat this process until we find such a row with the largest value. If there are still more than one rows after reaching the last column of *D*_*i*_, we randomly choose the edge polynomial corresponding to one of them. For example, in Fig. 5, both *Z*_4_ and *Z*_8_ has values in the first column. We choose one of them depending on their edge probability. If they have the same edge probability (i.e., *p*_4_=*p*_8_), we look at their values in the second column, where *Z*_4_ should be selected.

At the *i*th iteration, assume that we pick the edge polynomial *Z*_*r*_. We update $G_{k}^{i}$ for the next iteration by removing the node *v*_*r*_ from $G_{k}^{i}$ along with all of its incident edges.

### Overcoming memory bottleneck

The number of terms of the x-polynomial can grow exponentially, particularly when collapsation does not take place. This quickly leads to memory bottleneck especially for dense overlap graphs. In this section, we present a recursive strategy to overcome this bottleneck.

**Fig. 6:**
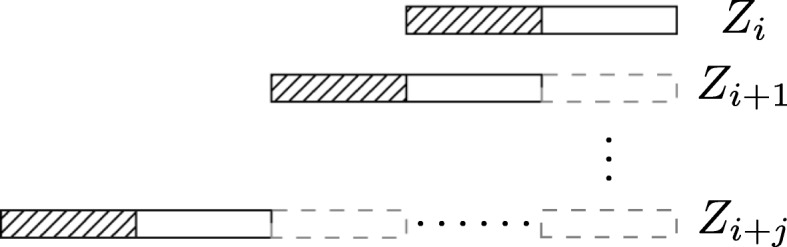
The change of the size of the polynomial terms. Bar with solid line: new generated polynomial terms after multiplying the current edge polynomial; bar with dashed line: deferred polynomial terms; shaded bar: polynomial terms that are chosen to multiply

The main idea behind this strategy is as follows. Given a new edge polynomial, we multiply it with only a subset of the current x-polynomial terms, while deferring others. After completing the multiplication of all edge polynomials, we multiply the deferred polynomial terms using *last-in, first-out* policy. Figure 6 depicts this idea. Here, the shaded bar represents the subset of terms of the current x-polynomial to be multiplied with the next edge polynomial (e.g., *Z*_*i*+1_). Let us denote the number of terms in this subset with *N*_1_ and the number of deferred terms with *N*_2_ prior to multiplying with *Z*_*i*+1_. After multiplication with *Z*_*i*+1_, the number of terms become 2*N*_1_+*N*_2_. Repeating this process, after multiplying *j* edge polynomials, we have only (*j*+1)*N*_1_+*N*_2_ terms. Notice that this is a dramatic improvement over the original algorithm which creates 2^*j*^(*N*_1_+*N*_2_) terms. The number of deferred terms is governed by the amount of available memory. That is we choose *N*_1_ as large as possible while the maximum term count ((*j*+1)*N*_1_+*N*_2_) remains less than available memory.

## Results and discussion

In this section, we experimentally evaluate the performance of ProMotE on synthetically generated (“Evaluation on synthetic networks section”) and real networks (“Evaluation on cancer networks” to “Evaluation on neurodegenerative disease networks section”). We run our experiments on a Linux server which has AMD Opteron 24 core processors (up to 1.4GHZ) and 32GB memory.

### Evaluation on synthetic networks

#### Running time evaluation

**Fig. 7:**
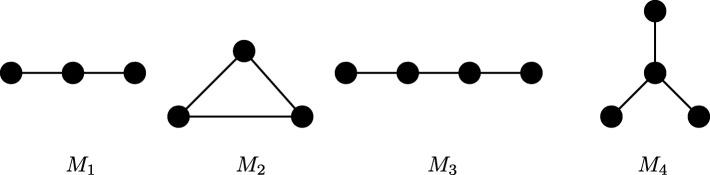
The four motifs with two and three edges

**Fig. 8:**
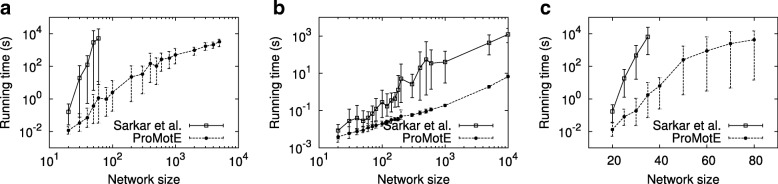
Running time on synthetic networks generated by ER, WS, and BA models respectively. The running times are reported in seconds and represented in log-scale. **a** ER model **b** WS model **c** BA model

The key computational challenge we aim to tackle in this paper is to scale to large network sizes while existing methods fail. Here, we evaluate how well we achieved this goal. We compare the running time of ProMotE to that of Sarkar et al. [24] as this is the most recent and most efficient algorithm in the literature to the best of our knowledge. As these two methods have the similar expected motif count, we do not report their motif count. We test both methods on synthetic networks. We generate random networks with growing number of nodes using three random network models, namely Erdős Rényi (ER) [25], Watts Strogatz (WS) [26] and Barabási-Albert (BA) [27] models. We synthetically assign a probability value generated from the uniform distribution in the interval (0,1) to each edge of networks. We set the average node degree to two. For each number of nodes, we repeat the experiments for 20 random networks. We measure the total running time for four motif patterns (see Fig. 7). These motifs are also used in the study by Sarkar et al. [24]. To avoid the potential outlier, for the 20 networks of each network size, we exclude the two networks with largest running time, and another two with the smallest running time. For the remaining networks, we report the average, minimum and maximum values. For each method, we report the running time on networks if the algorithm completes in less than 10,000 seconds. Figure 8 presents the results.

We observe that our method is several orders of magnitude faster than Sarkar et al. for all parameter settings. As the network size increases, the gap between the running times of two methods grows. This indicates that our optimization strategies are greatly helpful on large networks. The advantage of ProMotE stands out the most for the networks generated using ER and BA models. For instance, on networks generated using ER model (see Fig. 8a), ProMotE successfully scales to massive network sizes (i.e., thousands of nodes). On the other hand, Sarkar et al. fails to run beyond 100 nodes. This implies that ProMotE is not only practical but it is also essential to study real networks, as the size of real networks is often large. Furthermore, we observe that the topological characteristics of the network greatly affect the cost of counting independent motifs. For the same network size, we observe a dramatic increase in running time as we move from WS model to ER model and then to BA model. ER model generates networks with a small average shortest path length along with a small clustering coefficient. WS model however builds networks with both short average paths and strong clustering coefficients. Thus, networks generated by ER model are more likely to have isolated nodes, which in turn make the remaining connected components much denser than those in networks generated by WS model. Dense networks however result in more computation. As a result, ProMotE runs much faster on networks of WS model. For the BA model, it constructs the so called scale-free networks characterized by a highly heterogeneous degree distribution, which follows power law. Thus, there exist more hub nodes in networks of BA model, which result in more overlapping motif embeddings. As a result, the overlap graphs of networks of BA model are much more complicated, which in turn introduce a large quantity of computation. Recall that the BA model generates scale free networks. Thus it is expected to generate networks that resemble real data better than the other two models. This indicates that counting independent motifs in real networks is a challenging problem, and ProMotE is needed to study them in practical time.

#### Comparison against the literature

In the literature, current approaches to the probabilistic networks often transform probabilistic networks to deterministic networks first, and then apply methods for deterministic networks. These approaches include ignoring probability values [28, 29], considering edges with probability values above a given threshold [30], and sampling the probabilistic network by doing a Bernoulli trial with probability *p*_*i*_ for each edge *e*_*i*_ [31]. For simplicity, we denote these three approaches with *binary, threshold* and *sampling* method respectively. For each of these method, after transforming probabilistic networks to deterministic networks, we apply the method in the literature [19] to find the motif count, which heuristically selects motifs with the least number of overlapping embeddings. As these methods are not specifically devised to solve the problem in this paper and they finally work on deterministic networks, the performance of ProMotE in terms of running time is significantly worse than these methods. As a result, we compare our method against these methods only in terms of expected motif count.

**Fig. 9:**
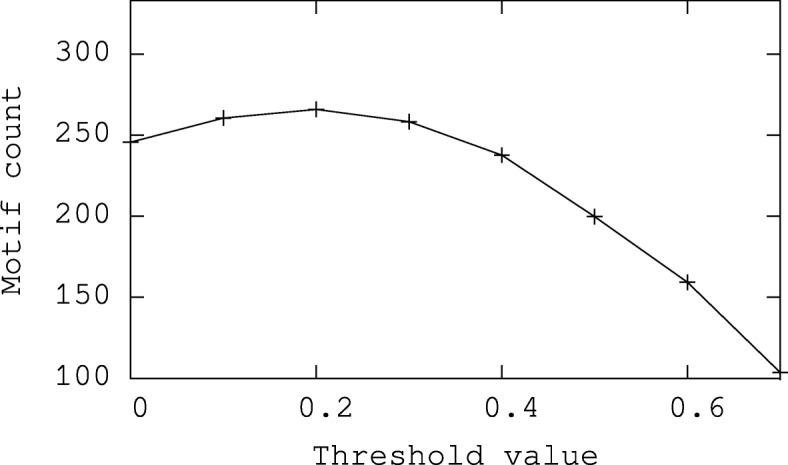
The expected motif count of our method and threshold method on synthetic networks

Recall that the threshold method maintains the set of edges with probability value above a given threshold value and removes the remaining edges. Thus, the outcome of this method depends on the threshold value. Here, we first evaluate the performance of the threshold method for varying values of threshold. We run our experiment on synthetic networks with WS model of various network sizes, 1000, 5000 and 10000. In particular, for each network size, we repeat the experiments for 20 random networks. We vary the threshold value from 0 to 0.7 at increments of 0.1. For each threshold value setting, we run experiment on all generated networks and report the average motif count. Figure 9 plots the results.

We observe that the motif count of the threshold method first grows with the increasing threshold value. It then falls sharply. It obtains the peak value when the threshold is 0.2. This is possibly because too small/large threshold leads to the retaining/removing most of the edges of the network. Either case may make the threshold method underutilize the information available in the interaction probabilities. As a result, a suitable threshold value is necessary for the threshold method. However, the varying distribution of edge probabilities of different probabilistic networks makes it is difficult to set a fixed threshold value for the threshold method. In the rest of our experiments, we fix the threshold value of the threshold method to 0.2 as it obtains the best value on the average across a broad spectrum of parameter settings.

**Fig. 10:**
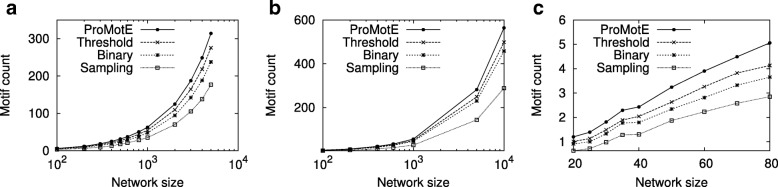
Expected motif count on synthetic networks generated by ER, WS, and BA models respectively. **a** ER model **b** WS model **c** BA model

Next, we compare our method with binary, threshold and sampling methods. We test these methods on synthetic networks with different network models of various network sizes. To ensure the reliability of our results, for each parameter, we conduct experiments on 20 different networks and report the average. Specifically, for sampling method, as each running on the same network may have the different value, to get a reliable result, we run 10 times on each network and report the average. Figure 10 reports the result. Our results demonstrate that our method has the highest motif count for all network sizes with various network models. Moreover, the gap between ProMotE and other methods increases with the growing number of nodes. This is very promising since we expect to have more number of nodes in real networks. For instance, on the network with WS network model of size 10,000, ProMotE has 13.2 to 95.4% more motif counts than other methods. The threshold method achieves the second best motif count. That said, it is worth noting that we give a positive bias towards the threshold method since we fix the threshold to the value which maximized its motif count in our previous experiments. Sampling method has overall worst performance across different network sizes. The reason behind is that finding a set of independent embeddings that yields the maximum expected number of occurrences in a probabilistic network is a nonlinear function. Although, the sampling approach can provide provable confidence intervals for estimating linear functions such as sum and average, it fails to do that for nonlinear functions such as counting independent motifs in probabilistic networks. Due to the nonlinear nature of our problem, a sampling approach is expected to produce inaccurate results. Furthermore, we observe that the expected motif count of all methods grows with the increasing network size. This is because the network with larger size is expected to have more motifs.

In summary, our method is very efficient and accurate on counting independent motif in probabilistic networks.

### Evaluation on cancer networks

In order to observe the performance of our method on real networks, we apply our algorithm on six cancer datasets from the MSigDB database [32] for *Homo Sapiens*. We extract genes from the C2:curated gene sets of MSigDB. We then feed each cancer gene set into the STRING database [16] to generate its interaction network. We use the gene co-expression values present in the STRING dataset for these networks to compute their interaction probabilities. Specifically, for a pair of nodes, the interaction probability between them is computed as the Pearson’s correlation coefficient. In the literature, there are also other ways to compute interaction probabilities. For example, Sharan et al. [33] addressed this problem by utilizing features like the volume of evidence present for the interaction, gene expression correlation, and network topology to learn the edge probabilities. Gabr et al. [34] used end-to-end signal reachability probabilities between pairs of genes to guide the computation of the edge probabilities. All these studies use transcriptional values in their computations in slightly different ways. Table 2 presents the dataset details for each cancer type. We measure independent motif counts for the four basic patterns listed in Fig. 7.

**Table 2:** Real networks from cancer dataset used in our experiments; number of nodes and edges, average node degree and clustering coefficient

Cancer	Nodes	Edges	Avg. degree	C. coefficient
Thyroid	39	43	2.21	0.773
Bladder	49	51	2.08	0.572
Endometrial	54	61	2.26	0.676
Lung	64	66	2.06	0.732
Colorectal	72	83	2.31	0.798
Pancreatic	80	87	2.17	0.791

**Fig. 11:**
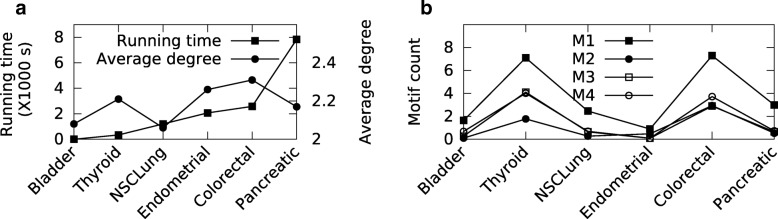
**a** Running time on cancer networks. The x-axis shows the cancer type. The y-axis on the left and right show the running time in seconds and average degree of each network respectively. **b** The $\mathcal {F}_{2}$ (i.e. number of non-overlapping instances) count of each of the four basic motifs in real network. The x-axis shows the cancer type. The y-axis shows the motif $\mathcal {F}_{2}$ count

Figure 11a presents the running time of ProMotE. In addition, we also plot the average degree of each network to display the effect of running time on network size. Our results demonstrate that ProMotE successfully identifies independent motifs in practical time (1.5 secs to less than 2.2 h) for all networks. It is worth noting that Sarkar et al.’s method does not scale to these network sizes.

Figure 11b shows the number of non-overlapping instances of the four basic patterns present in each of the cancer networks. We observe that the pattern M1 is the most abundant topology in all networks. This is expected as M1 is a subgraph of M2, M3, and M4. The other three motifs have similar counts. We also observe that the motif count does not necessarily grow with the network size. For instance Thyroid cancer has the least number of nodes and edges, yet it contains more non-overlapping motif instances than almost all the other networks in our dataset. This implies that the topology of the network governs the motif distribution. Recall that the running time of ProMotE tends to grow with network size on the cancer networks (see Fig. 11a). This implies that the running time of ProMotE does not strongly depend on the independent motif count as well.

### Evaluation on neurodegenerative disease networks

Next, we evaluate ProMotE on three neurodegenerative disease networks; Alzheimer’s, Huntington’s and Parkinson’s disease, hereafter referred to as AD, HD and PD respectively. We obtain this dataset from the MSigDB database similar to the cancer networks (see “Evaluation on cancer networks section”). Table 3 lists the dataset details. One major difference between this and the cancer dataset is that the neurodegenerative networks are both larger in size and average degree. It is worth noting that the AD network is a subgraph of that of HD network. Also, all three networks share substantial amount of edges (details later in this section). We focus on motif M2 (the loop pattern) in this experiment.

**Table 3:** Real networks from neurodegenerative dataset used in our experiment, the disease name, numbers of nodes and edges, average node degree and clustering coefficient

Disease	Nodes	Edges	Avg. degree	C. coefficient
AD	162	737	9.1	0.876
HD	177	756	8.54	0.845
PD	125	751	12	0.827

AD, HD and PD correspond to Alzheimer’s, Huntington’s and Parkinson’s disease, respectively

Table 4 shows the results. We observed that AD and HD networks yield the same number of embeddings and $\mathcal {F}_{2}$ count. Totally, 37 to 56% of the genes participate in at least one motif embedding. PD network has the largest motif count and fraction of genes in motif embeddings. Such large motif count indicates that these networks are organized largely as a combination of small loops. Finally, our method completed counting motifs in slightly over half an hour per network. This demonstrates that our method scales up to very large network sizes and densities.

**Table 4:** Results on real networks from neurodegenerative dataset used in our experiment, the disease name, unique number of genes in the result set, number of embeddings, $\mathcal {F}_{2}$ measure and the running time

Disease	Unique Genes	Embeddings	$\mathcal {F}_{2}$	Time (s)
AD	67	228	87.2108	1984.74
HD	67	228	87.2108	2006.99
PD	70	234	88.6849	2019.60

**Fig. 12:**
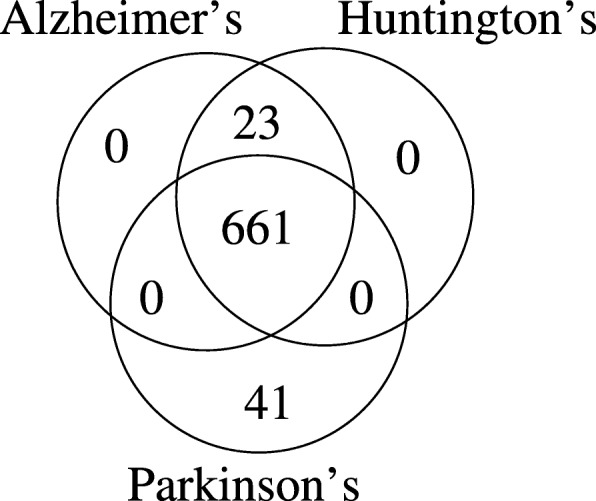
The number of common edges present in the three disease networks for independent embeddings of the triangle motif pattern

Next, we take a closer look at the distribution of the shared edges, which appear in a motif embedding, across different diseases. Figure 12 presents the distribution. We observe that the edges present in AD and HD are exactly the same. PD network however deviates from the other two by about 6%. This implies that all three diseases possibly are governed by very similar processes, with PD having slight variations.

Next, we focus on the statistical significance of the biological processes and molecular functions of the genes that comprise the result set for the three neurodegenerative diseases for M2 pattern. To do this, we perform gene ontology analysis using PANTHER [35]. For each network, we use all the genes appearing in an embedding of M2. We filter the Gene Ontology (GO) terms with *p*-value above 0.025. We then search each of the remaining GO term in PubMed with the disease corresponding to that network (i.e., AD, HD, or PD). Table 5 shows the results for which at least one reference publication exists.

**Table 5:** Gene ontology analysis of the genes appearing in the independent embeddings of the triangle pattern in the three disease networks with publications, and the diseases associated with the ontology terms

Category	GO ID	Function name	*p*-value	Reference	Disease
	0006120	mitochondrial electron transport, NADH to ubiquinone	2.65E-58	Perier and Vila [36]	PD
	0042776	mitochondrial ATP synthesis coupled proton transport	1.43E-20	VanDuyn et al. [37]	AD,PD
Biological	0006123	mitochondrial electron transport, cytochrome c to oxygen	4.66E-20	Fiskum et al. [38]	PD
Process	0006122	mitochondrial electron transport, ubiquinol to cytochrome c	5.13E-19	Kim et al. [39]	AD
	0006099	tricarboxylic acid cycle	2.43E-02	Shi et al. [40]	AD
	0003954	NADH dehydrogenase activity	9.19E-53	Zubenko et al. [41]	AD
	0004129	cytochrome-c oxidase activity	1.4E-18	Cardoso et al. [42]	AD
Molecular	0008121	ubiquinol-cytochrome-c reductase activity	9.53E-14	Liang et al. [43]	AD
Function	0000104	succinate dehydrogenase activity	6.42E-06	Fattoretti et al. [44]	AD,HD
	0048038	quinone binding	8.33E-04	Wang et al. [45]	AD,PD
	0051537	2 iron, 2 sulfur cluster binding	4.56E-03	Isaya [46]	PD

Our results demonstrate that ProMotE identifies disease specific functional properties. For instance, mitochondria often referred to as the *power house of the cell* is responsible for many important cellular functions especially neuronal viability. Therefore, aberrations in mitochondrial processes have potential to lead to neuronal disorder. Four distinct pathways carry out mental processing for brain metabolism, two of them being tricarboxylic acid cycle (TCA) and electron transport chain (the other two are glycolysis, pentose shunt). These pathways are affected by direct modification of the enzymes or alterations at the gene expression level. TCA is responsible for producing reducing equivalents in the form of reduced nicotinamide adenine dinucleotide (NADH) and reduced flavin adenine dinucleotide (FADH2). Altered activities of the TCA cycle enzymes result in imbalance which is associated with AD-related changes in metabolism.

Another important function is the production of adenosine triphosphate (ATP) through the combined effects of TCA and the respiratory chain, also known as electron transport chain. The respiratory chain requires two electron carriers: ubiquinone/coenzyme Q and cytochrome c. Also, it consists of five protein complexes: NADH dehydrogenase-ubiquinone oxidoreductase (complex I), succinate dehydrogenase-ubiquinone oxidoreductase (complex II), ubiquinone-cytochrome c oxidoreductase (complex III), cytochrome c oxidase (complex IV), and ATP synthase (complex V). Production of ATP involves two coordinated mechanisms: electrons received from energy substrates such as NADH are transported through the mitochondrial complexes towards molecular oxygen, producing water; at the same time electrochemical gradient is generated by driving protons across the mitochondrial inner membrane by I, III, and IV protein complexes. Finally, ATP is produced by the accumulation of these protons into the matrix with the help of complex V (ATP synthase). Altered mitochondrial respiration, especially at the level of complex I thus associates with PD.

## Conclusions

In this paper, we developed ProMotE, an efficient method to count non-overlapping motif instances in probabilistic networks. This method uses a polynomial model to capture the dependencies between overlapping embeddings. We proposed three strategies to avoid computation of loss value, to expedite collapsation of polynomial terms, and to overcome the memory bottleneck faced when applied to large networks. Our experiments on both synthetic and real networks demonstrate that our method scales to large networks and identifies the key functional characteristics of cancer and disease phenotypes.

## Abbreviations

AD: Alzheimer’s disease
ATP: Adenosine triphosphate
BA: Barabási-Albert
ER: Erdős Rényi
FADH2: Reduced flavin adenine dinucleotide
HD: Huntington’s disease
NADH: Reduced nicotinamide adenine dinucleotide
ProMotE: Probabilistic motif embedding
PD: Parkinson’s disease
TCA: Tricarboxylic acid cycle
WS: Watts Strogatz
